# Seasonal and altitudinal dynamics in secondary metabolite composition of *Commelina* forage species in Konso zone, southern Ethiopia

**DOI:** 10.1371/journal.pone.0314358

**Published:** 2024-11-26

**Authors:** Kebede Gelgelo, Yisehak Kechero, Dereje Andualem

**Affiliations:** 1 Department of Animal Sciences, College of Agricultural Sciences, Arba Minch University, Arba Minch, Ethiopia; 2 Department of Animal and Range Sciences, College of Agriculture and Natural Resources, Dilla University, Dilla, Ethiopia; Makerere University, UGANDA

## Abstract

Exploring the type and amounts of the secondary metabolites (SMs) in a given fodder species was considered as a meaningful act for safe and profitable utilization of that particular feedstuff in the livestock industry. This study was conducted in the Konso zone, southern Ethiopia, to explore the secondary metabolite composition of *Commelina* species in two seasons and at two different altitudes. Samples were collected from the two altitudes and seasons. A completely randomized design was used in a factorial arrangement (five species (*C*. *benghalensis*, *C*. *imberbis*, *C*. *diffusa*, *C*. *albescens*, and *C*. *africana)*, two altitudes, and two seasons) with three repetitions per treatment. The SM contents of the *Commelina* species were reasonably influenced by both seasonal and altitudinal changes. The mean alkaloid (3.67%), total phenols (9.76 mg GAE/g), flavonoid (3.81 mg CE/g) and condensed tannin (1.10 mg CE/g) values for the herb species in wet season inclined (p < 0.001) to 7.02%, 14.07 mg GAE/g, 7.68 mg CE/g and 2.38 mg CE/g, respectively, in dry season. The wet season saponin concentration of the species (2.65 g/Kg) significantly decreased (p < 0.001) to 1.28 g/Kg in the dry season. Similarly, the lowland saponin (2.26 g/Kg), alkaloid (3.70%), total phenols (10.89 mg GAE/g), flavonoid (4.71 mg CE/g), and condensed tannin (0.98 mg CE/g) contents were increased (p < 0.01) to 3.03 g/Kg, 5.47%, 13.61 mg GAE/g, 6.37 mg CE/g, and 1.81 mg CE/g, respectively, in the midlands. Alkaloids, total phenols, flavonoids and condensed tannin concentrations showed positive correlations with each other (P<0.05) and with seasonal (P<0.001) and altitudinal changes (P<0.001) as well. The findings of this study suggested that the SM concentrations of *Commelina* species were within the limits tolerable for ruminants. In conclusion, *Commelina* species could serve as a safe and beneficial forage herb to boost nutrient intake, improve nutrient use efficiency and hinder methane emissions, for animals consuming them, in areas where they are available in abundance.

## Introduction

Plants, from their germination through their growth and development till the end of their life span, stay in a complex and netted relationship with their surrounding environment, continually interacting with both the biotic and abiotic elements of their living habitat. In the course of this interaction, these factors impose multitudinal effects. In response to these effects, plants react by producing biochemicals called secondary metabolites (SMs) to escape from stress conditions and sustain their lives [1–3]. These SMs, also called phytochemicals, are produced as outputs of primary metabolism and serve multiple functions for plants. Typically, they have no role in primary metabolic activities (photosynthesis, growth, respiration and excretion), but are important for adaptation to stress conditions and defense mechanisms. Therefore, SMs were considered as biological molecules mediating the chemical interaction between plants and their surrounding environment [4].

SMs are present in the leaves, stems, roots, and flowers of different plants ranging from herbaceous plants to woody trees and grasses to leguminous plants [5, 6]. The importance of secondary metabolites as defending agents counter to external influences is not only limited to plants but also extended to human beings providing high medicinal value against various ailments [7]. In the context of animal nutrition, the biological effect of SMs takes on two dimensions. When consumed at safe doses, the chemical constituents provide multiple nutritional and health benefits [8, 9]. On the contrary, they limit digestibility and availability of valuable nutrients, diminish animal productivity and may cause toxicity and death in severe cases in periods of scarcity or confinement when they are consumed by animals above the tolerable threshold [10]. According to Kemboi et al., [11], the balance between beneficial and lethal effects of SMs depends on their time of exposure, concentration, biochemical structure, and interaction with other dietary constituents. Therefore, exploring the type and quantity of secondary metabolites (SMs) in a given fodder species was considered as a meaningful act for safe and profitable utilization of that particular feedstuff in the livestock industry.

*Commelina* species were known to have highly broader patterns of distribution. Those members like *C*. *benghalensis* and *C*. *imberbis* adapt wide range of habitats worldwide [12] where as others like *C*. *albiflora Faden* have narrow range distribution or even specific habitats as it occurs only in Western Kenya, East Africa [13]. East Africa harbors about 51 species out of which 17 species were confirmed in Ethiopia. *C.diffusa* mostly prefers water lodged areas and river sides and its abundance diminishes gradually moving far away from water bodies. *C*. *albescens* and *C*. *africana* grow abundantly at altitudes ranging from 300 to 1,700 masl, but it is difficult to find these species growing above 2,000 masl [14, 15]. *Commelina* species were known for their feed use for many livestock species and are being used widely by livestock keepers of the Konso community. Livestock keepers in the study area allow *Commelina* species to grow as an alley crop on arable land. The species growing in harmony and socially with food crops provide plenty of multiple advantages for agricultural productivity in the area besides animal feed usage [14]. It was well established that dairy cattle require a minimum crude protein (CP) of 7.5% CP for maintenance [16], 11.3% and 12% for growth and lactation respectively [17]. So, with average CP values ranging between 17.59–17.71%, *Commelina* species could be used as supplement to support growth and production dairy operations in areas where available in abundance [18]. In support of this, Kebede et al. [19] argued that, with promising profit, *Commelina* species could be used as a supplement to improve the animals’ nutrient uptake and growth efficiency. Even though few reports were available on feed usage potential of *Commelina* species, the nature and quantities of secondary metabolites contained in different *Commelina* species has got little consideration and is not well narrated among scientists. Besides, it was well recognized that, the presence of SMs above a certain limit in a given fodder species inhibits the availability of other soluble nutrients contained in that particular feed staff to animals consuming it [10]. So, for better description of the nutritive potential and safe usage of *Commelina* species in the feed sector, examining the types and amounts of secondary metabolites in the species was highly desirable. The type and amounts of the SMs were reported to be influenced by many factors viz. genetic differences, variability in environmental conditions associated with seasonal and altitudinal changes (fluctuating temperatures, UV radiation, salinity, oxygen concentration, and wind velocity [20, 21]. Therefore, this study hypothesizes that with changing seasons and altitudes, the secondary metabolite composition of *Commelina* species could vary significantly. And thus, the aim of this research work was to investigate the seasonal and altitudinal dynamics in secondary metabolite composition of five *Commelina* forage spp.; *Commelina benghalensis*, *Commelina imberbis*, *Commelina diffusa*, *Commelina albescens*, and *Commelina africana* available in Konso zone, southern Ethiopia.

## Material and methods

### Description of the study area

The study was conducted at Konso zone of southern Ethiopia. The study site is located about 595 km south of Addis Ababa, the capital city of Ethiopia, and 360 km south of Hawassa, capital of the southern nation nationalities and people’s regional state (SNNPRS). Geographically, it is located at 5010’0’’ to 5040’0’’N latitude and 3700’0’’ to 37045’0’’ E longitude. The total land area of the Zone is 2016.24 Km2. The altitude of the area is between 501–2000 meters above sea level. The main agro-ecological divisions of Konso are 70% lowland (Kola) and 30% account tropical midland (Weinadega). The mean annual temperature of the zone ranges between 17.6–27.50°C. The mean annual rainfall ranges between 601–1200 mm [22].

### Sample collection

Samples of all *Commelina* species were collected from both the low and mid altitudes of the study area at dry and wet seasons of the year. Dry season samples were collected at the mid of drier months, from mid-June to mid-July 2020 and mid-December 2020 to mid-January 2021 while wet season samples were collected at the blooming stage, from mid-September to mid-October 2020 and end-February to end-March 2021. The samples were then mixed thoroughly per altitude per season where three subsamples were taken for each species and brought to the animal nutrition laboratory for detailed investigation of secondary metabolite composition. The collected samples were partially dried (at 65°C for 72 h) and ground to pass through 1 mm sieve mill, stored in airtight and moisture tight bags pending further analysis of secondary metabolites.

### Determination of the secondary metabolite composition

#### Total phenols

The total phenol content was estimated by the Folin-Ciocalteu method [23]. 0.1 mL of solution (1 mg/mL) was mixed with 1 mL of diluted Folin-Ciocalteu reagent. The mixture was left for 5 min and then 1 mL of sodium carbonate (7.5g/100mL solution) was added. After incubation at 25°C for 90 min, the absorbance of the resulting blue color was measured at 765 nm using a UV-visible spectrophotometer (HACH LANGE DR 5000). The concentration of total phenolic content was estimated from the gallic acid (1–100 μg/mL) calibration curve (y = 0.023x - 0.032, R2 = 0.98) and the results were expressed as milligrams of gallic acid equivalent per 100 grams (mg GAE/g dried extract).

#### Flavonoid

The total flavonoid content was estimated using the method of Belachew *et al*. [24]. One milliliter of the solution (1 mg/mL) was diluted with distilled water to a volume of 2.25 mL and 75 μL 5% NaNO2 was added to the mixture. After 6 min, 150 μL 10% AlCl3 and then after 5 min, 1 mL 1 M NaOH were added to the reaction mixture. Finally, after mixing immediately the absorbance of the pink color was determined at 510 nm versus prepared water blank. A standard curve (y = 0.013x + 0.027, R2 = 0.97) was prepared using catechin (5–1000 μg/mL). The concentration was expressed in milligrams of catechin equivalents per 100 grams (mg CE/g dried extract).

#### Condensed tannin

The condensed tannin content was performed according to the method described by Chew *et al*. [25]. 0.5 mL undiluted crude extract was first mixed with 3 mL of vanillin reagent (4%, w/v, in absolute methanol), followed by addition of 1.5 mL of concentrated HCl (37%). Mixture was stored in a dark environment at room temperature for 15 min. Blanks were prepared by replacing 0.5 mL of undiluted crude extract with 0.5 mL of deionized water. The absorbance of the mixture was measured at 500 nm against the blank using a UV light spectrometer (JENWEY, 6300, Switzerland). The absorbance of the blank was subtracted from the absorbance of the corresponding vanillin-contain sample. A standard curve has been constructed (Absorbance vs Catechin) after correcting for blank and the linear portions of the curve were extrapolated to produce the standard curve. Catechin was used for calibration of the standard curve (y = 0.005x + 0.0231, R2 = 0.99) and the results were expressed as mg catechin equivalent per 100 g dry weight sample (mg CE/ g dried extract).

#### Alkaloids

Exactly 1g of the plant sample was extracted repeatedly with 10 ml of 80% aqueous methanol at room temperature. The whole solution was filtered through whatman filter paper No 42. The filtrate was later transported into a container and evaporated into dryness over a water bath and weighed to a constant weight [26]. Then the yield was expressed in terms of % (w/w).

#### Saponins

The samples were ground and 2 g of each was put into a conical flask and 10 ml of 20% aqueous ethanol was added. The samples were heated in a hot water bath for 4 h with continuous stirring at about 55°C. The mixture was filtered and the residue re-extracted with another 20ml of 20% ethanol. The combined extracts were concentrated over water bath at about 90°C. The concentrate was transferred into a 250 ml separating funnel and 10 ml of diethyl ether was added and shaken strongly. The aqueous layer was recovered while the ether layer was cast-off. The purification process was repeated. 6 ml of n-butanol was added. The combined n-butanol extracts were washed twice with 1ml of 5% aqueous sodium chloride. The residual solution was heated in a water bath. After evaporation the samples were dried in the oven to a constant weight; the saponin content was calculated (%, w/w) [26].

#### Data analysis

The five species, two altitudes, and two seasons were arranged in a 5×2×2 factorial arrangements with three replications for each treatment in each site. The data analysis model is shown as follows:

Yijk=μ+αi+βj+δk+αβij+αδik+Σijk;

where, Yijk, response variable; μ, overall mean; αi, species effect; βj, effect of altitude (low and mid); δk, effect of the season (dry and wet); αβij, effect of the species X altitude interaction; αδij, effect of the species X season interaction; Σijk, random error.

The data were analyzed using the PROC GLM of SAS [27]. Means were separated using the Duncan’s multiple range test. The level of significance was determined at (P < 0.05). Pearson’s correlation was employed to examine the nature and strength of relationships between the season, altitude and the secondary metabolites investigated.

#### Ethical clearance statement

To conduct the present study entitled “Seasonal and Altitudinal Dynamics in Secondary Metabolite Composition of Commelina Forage Species in Konso Zone, Southern Ethiopia” permission was received from Arba Minch University Animal Research Ethics Review Committee (Approval Number AMU/AREC/2/2016).

## Results

### Seasonal variability in the secondary metabolite content of *Commelina* species

All of the secondary metabolites in *Commelina* species were significantly (p < 0.001) influenced by season, but the pattern of seasonal variability was not consistent for all of the parameters (Table 1). Except saponin, the contents of the rest parameters were higher in the dry season (P<0.001) than in the wet season. Between species variability was also significant (P<0.05) for all of the parameters except flavonoid composition (P>0.05). The two-way ANOVA indicates that the species x season interaction effect was insignificant (P>0.05) for all of the secondary metabolites (Table 3).

**Table 1:** Seasonal variability in the secondary metabolite content of *Commelina* species.

Season	Species	Saponin (g/Kg)	Alkaloid (%)	Total Phenols (mg GAE/g)	Flavonoid (mg CE/g)	Condensed Tannin (mg CE/g)
Wet season	*C.benghalensis*	3.11^b^	3.72^b^	11.78^a^	4.65^a^	0.89^ab^
	*C.imberbis*	2.22^bc^	2.01^c^	6.79^b^	2.85^b^	0.40^b^
	*C.diffusa*	5.07^a^	3.35^b^	10.73^ab^	4.62^a^	0.89^ab^
	*C.africana*	1.57^c^	4.25^ab^	10.12^ab^	4.04^a^	1.73^a^
	*C.albescence*	1.26^c^	5.04^a^	9.36^ab^	2.92^b^	1.58^a^
	Seasonal Mean	2.65^A^	3.67^B^	9.76^B^	3.81^B^	1.10^B^
	SEM	0.46	0.36	0.78	0.28	0.19
Dry Season	*C.benghalensis*	2.28^ab^	6.57^a^	15.78^a^	6.69^a^	2.45^b^
	*C.imberbis*	0.54^bc^	6.44^a^	14.46^ab^	7.37^a^	1.21^b^
	*C.diffusa*	2.66^a^	5.85^a^	3.96 ^ab^	7.07^a^	2.51^b^
	*C.africana*	0.17^c^	8.21^a^	13.05^b^	8.10^a^	3.94^a^
	*C.albescence*	0.76^bc^	8.03^a^	13.09^b^	9.15^a^	1.82^b^
	Seasonal Mean	1.28^B^	7.02^A^	14.07^A^	7.68^A^	2.38^A^
	SEM	0.36	0.45	0.47	0.54	0.34
Significance		***	*	*	***	**

* = P<0.05

** = P<0.01

***P<0.001

^a,b,c^Column means with different superscripts shows significant between species difference; ^A,B^Column means with different superscripts shows significant seasonal difference; mg CE milligram of catechin equivalent; mg GAE = milligram of gallic acid equivalent; SEM = Standard Error of the Mean

In the present study, saponin contents were in the range of 1.26–5.05g/Kg in the wet season, but dropped to 0.17–2.66 g/Kg in the dry season. In the wet season, the highest significant (P<0.001) saponin content was observed in *C.diffusa*, while *C.africana* and *C.albescence* had the least significant values comparable with each other and with that of *C.imberbis* also. In the dry season, the highest (P<0.001) saponin concentrations were recorded for *C.diffusa* and *C.benghalensis* while *C.africana* had the lowest record of the parameter though the values did not vary significantly from those of *C.imberbis* and *C.albescence*.

A meaningful increasing (p < 0.001) trend was observed from the wet season (3.67%) to the dry season (7.02%) in the alkaloid concentration of *Commelina* species. The highest wet season alkaloid values were recorded for *C*. *albescence* and *C*. *africana*, while *C*. *imberbis* had the least. In the dry season, regardless of numerical variations, all of the species contained statistically similar (p > 0.05) amounts of alkaloid among each other. The average total phenol values ranged from 6.79 to 11.78 mg GAE/g in the wet season, which augmented to 13.05 to 15.78% in the dry season. In the wet season, all of the *Commelina* species had comparable (P>0.05) total phenol values with the exception that, *C.benghalensis* had slightly higher (P<0.05) values relative to that of *C.imberbis*. Dry season observations also reveal that, all of the *Commelina* species had comparable (P>0.05) total phenol values with the exception that, *C.benghalensis* had slightly higher (P<0.05) values relative to those of *C.africana* and *C.albescence*.

**Fig 1:**
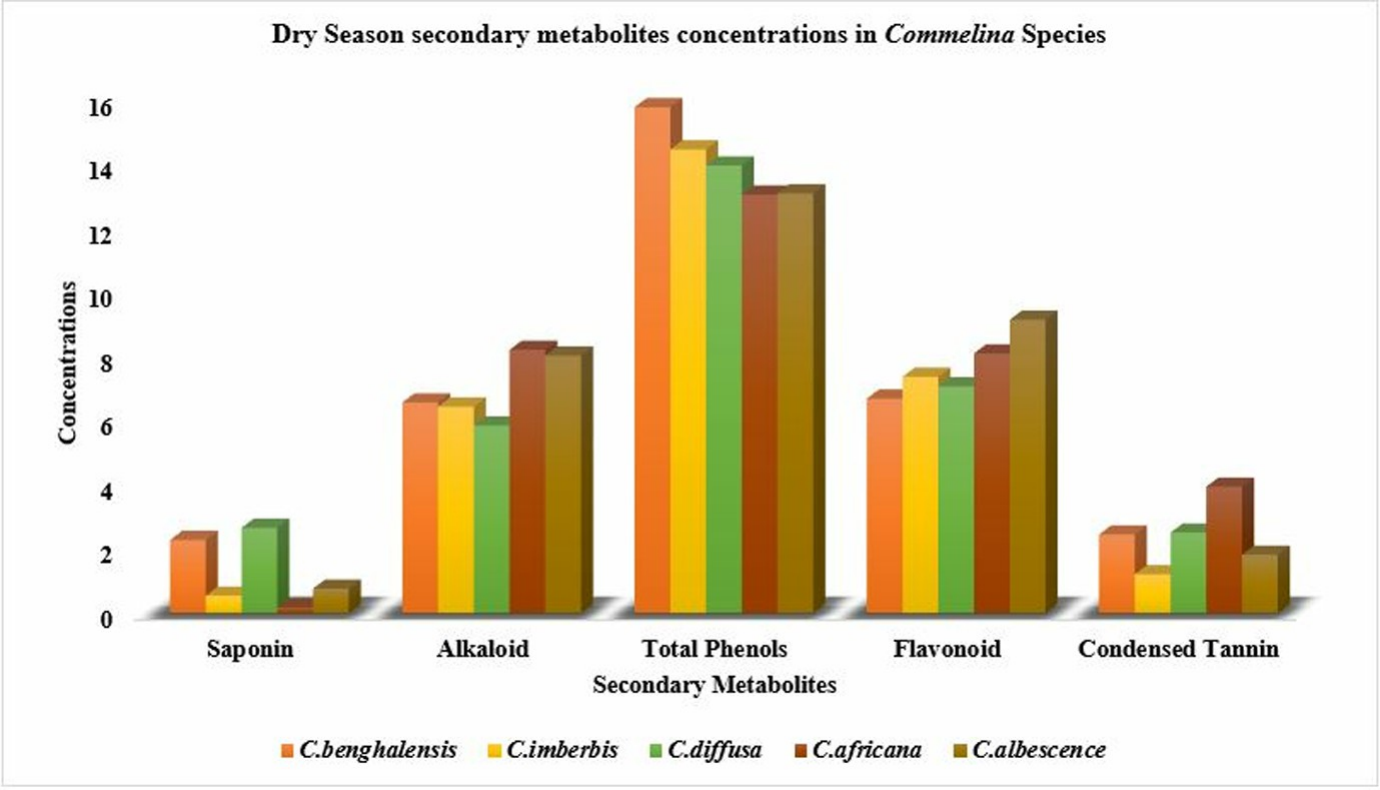
Composition of total phenols in milligram gallic acid equivalents per gram (mg GAE/g), flavonoid and tannin as catechin equivalents (mg CTE/g), saponin (g/Kg) and alkaloids (%) in different *Commelina* species in dry season in Konso zone, southern Ethiopia.

**Fig 2:**
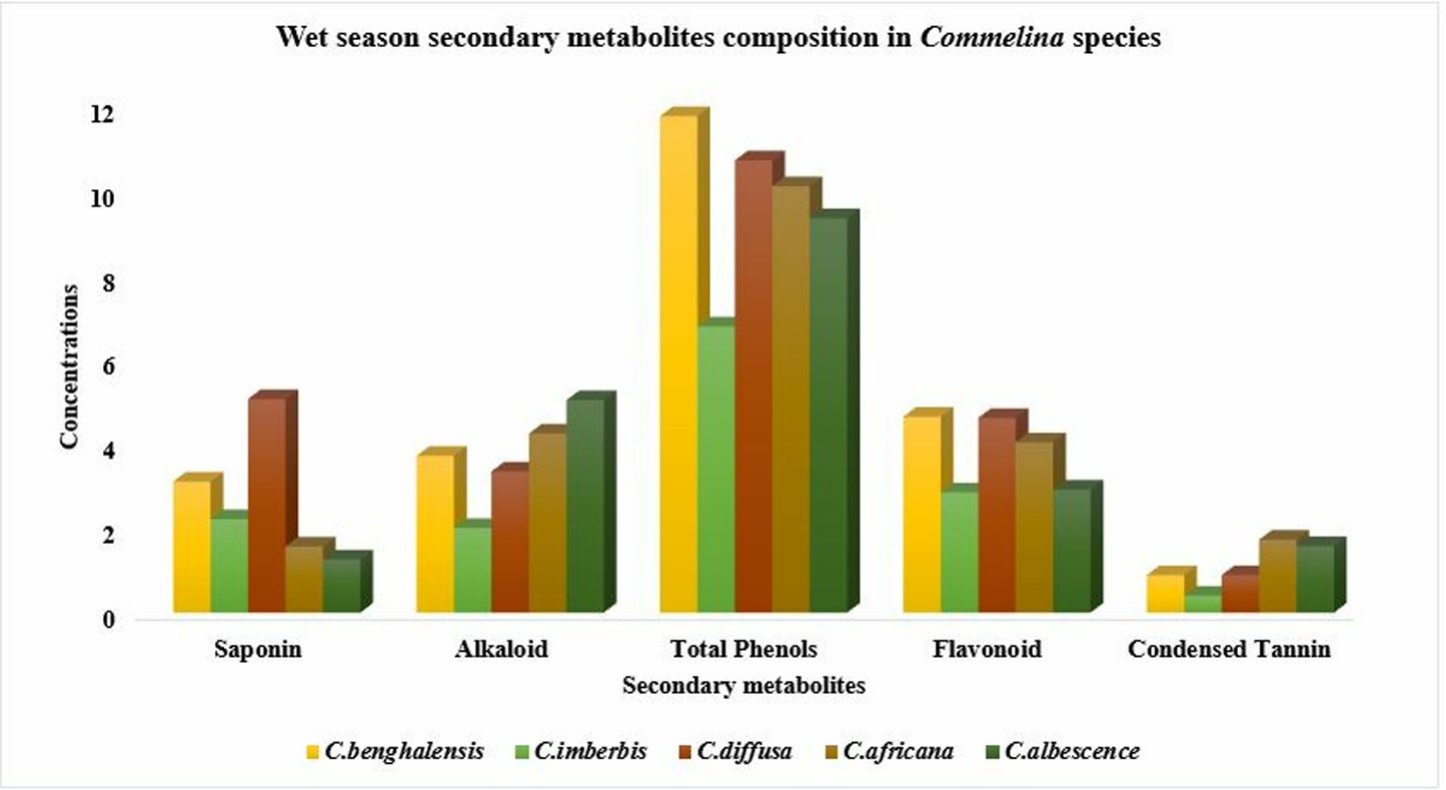
Composition of total phenols in milligram gallic acid equivalents per gram (mg GAE/g), flavonoid and tannin as catechin equivalents (mg CTE/g), saponin (g/Kg) and alkaloids (%) in different *Commelina* species in wet season in Konso zone, southern Ethiopia.

All of the *Commelina* species examined in this study do not show substantial differences (p > 0.05) in their dry season flavonoid concentrations (Table 1). On the contrary, the wet season flavonoid compositional difference was significant (p < 0.01) between species, with the least mean value recorded in *C*. *imberbis* and *C*. *albescence*, while all of the rest species had higher values comparable among each other. The mean condensed tannin values fluctuated between 0.40 and 1.73 mg CE/g during the wet season, and 1.21–3.94 mg CE/g during the dry season. Among-species variability was highly significant (p < 0.001) for both seasons, but the pattern of variability was inconsistent between seasons. In the wet season, *C.benghalensis* and *C.diffusa* had condensed tannin concentrations similar (P>0.05) with each other and with those of the rest *Commelina* species. But the records of *C.imberbis* were significantly lower than those of *C.africana* and *C.albescence*. Dry season records, in the present study, revealed that *C.africana* was the species with the highest (P<0.01) condensed tannin concentration, while all of the rest species had the least values comparable with each other. Among species, the variability pattern in secondary metabolite composition of *Commelina* species was illustrated in Fig 1 (Dry season patterns) and Fig 2 (Wet season patterns).

### Altitudinal variability in the secondary metabolite content of *Commelina* species

Table 2 shows the influence of altitude on the secondary metabolite composition of *Commelina* species in the Konso zone, southern Ethiopia. Altitude significantly (p < 0.01) influenced the concentration of all of the secondary metabolites in *Commelina* species. All of the secondary metabolites examined in this study exhibited a consistent pattern of variability between altitudes such that, progressing from lowlands to midlands, the secondary metabolite concentrations showed an increasing trend (P<0.0.01). Two-way ANOVA outputs revealed that altitude x species interaction was not significant (P>0.05) for all of the parameters investigated in this study (Table 3). On the other hand, the altitude x species interaction effect was highly significant (P<0.01) for all of the parameters except total phenols (P>0.05), whereas, season x species x altitude interaction was highly significant (P<0.01) for all of the secondary metabolites.

**Table 2:** Altitudinal variability in the secondary metabolite content of *Commelina* species.

Altitude	Species	Saponin (g/kg)	Alkaloid (%)	Total Phenols (mg GAE/g)	Flavonoid (mg CE/g)	Condensed Tannin (mg CE/g)
Lowland	*C.benghalensis*	2.27^b^	4.23^a^	11.98^a^	5.46^a^	1.29^a^
	*C.imberbis*	1.14^c^	3.26^a^	9.73^b^	3.68^b^	0.35^b^
	*C.diffusa*	3.37^a^	3.63^a^	10.96^ab^	4.99^a^	1.29^a^
	Altitudinal Mean	2.26^B^	3.70^B^	10.89^B^	4.71^B^	0.98^B^
	SEM	0.41	0.39	0.90	0.38	0.23
Midland	*C.benghalensis*	3.11^b^	6.16^a^	15.59^a^	5.88^a^	2.05^a^
	*C.imberbis*	1.61^c^	4.94^b^	11.51^a^	6.54^a^	1.26^b^
	*C.diffusa*	4.37^a^	5.33^b^	13.73^a^	6.70^a^	2.11^a^
	Altitudinal Mean	3.03^A^	5.47^A^	13.61^A^	6.37^A^	1.81^A^
	SEM	0.41	0.62	0.98	0.72	0.28
Significance	Altitude	**	*	**	**	***

* = P<0.05

** = P<0.01

***P<0.001

^a,b,c^Column means with different superscripts shows significant between species difference; ^A,B^Column means with different superscripts shows significant seasonal difference; mg CE milligram of catechin equivalent; mg GAE = milligram of gallic acid equivalent; SEM = Standard Error of the Mean.

**Table 3:** ANOVA table for effect of season, species, altitude and their interaction on secondary metabolite composition of *Commelina* species.

Factors	DF	F Value	Total Phenols (mg GAE/g)	Flavonoid (mg CE/g)	Alkaloids (%)	Saponins (g/Kg)	Condensed Tannins (mg CE/g)
Season	1	544.09	***	***	***	***	***
Species	4	2491.88	**	Ns	*	***	**
Altitude	1	1920.59	**	**	***	***	***
Season x Species	4	191.31	Ns	Ns	ns	Ns	Ns
Altitude x Species	2	22.32	Ns	Ns	ns	Ns	Ns
Season x Altitude	1	43.70	Ns	***	***	***	**
Season x Species x Altitude	2	33.05	**	***	***	***	***

* = P<0.05

** = P<0.01

***P<0.001

mg CE milligram of catechin equivalent; mg GAE = milligram of gallic acid equivalent DF = Degree of freedom; ns = non-significant (P>0.05)

The saponin content of *Commelina* species showed a consistent variability pattern with changing altitudes. The values ranged from 1.14 to 3.37 g/Kg in the lowland, and from 1.61 to 4.37 g/Kg in the midland, with the highest (p < 0.01) records noted for *C*. *diffusa*, the intermediary value for *C*. *benghalensis*, and the least for *C*. *imberbis* at both altitudes. Alkaloid concentrations of the *Commelina* species oscillated between 3.26–4.23% in the lowlands and 4.94–6.19% in the midlands of the study area (Table 2). In the lowlands, regardless of numerical variations, all of the species contained statistically similar (*p* > 0.05) amounts of alkaloid to each other. However, midland alkaloid contents significantly (*p* < 0.05) differed among species, with lower records equally observed for *C*. *diffusa* and *C*. *imberbis*, and higher values for *C*. *benghalensis*.

A significant increasing trend was observed in the total phenol contents of *Commelina* species moving from lowlands (10.89 mg GAE/g) to midlands (13.61 mg GAE/g) of the study area. In the midlands, all of the *Commelina* species examined in this study had similar (p > 0.05) total phenol concentrations. In the lowlands, the total phenol contents significantly vary between species such that, the values of *C.benghalensis* were higher (P<0.01) than to that of *C.imberbis* but comparable with records of *C.diffusa*. In the same way, flavonoid concentrations did not show significant variability (P>0.05) between species in the midlands. But in the lowlands, *C.benghalensis* and *C.diffusa* had comparable flavonoid concentrations higher (P<0.05) than that of *C.imberbis*.

**Fig 3:**
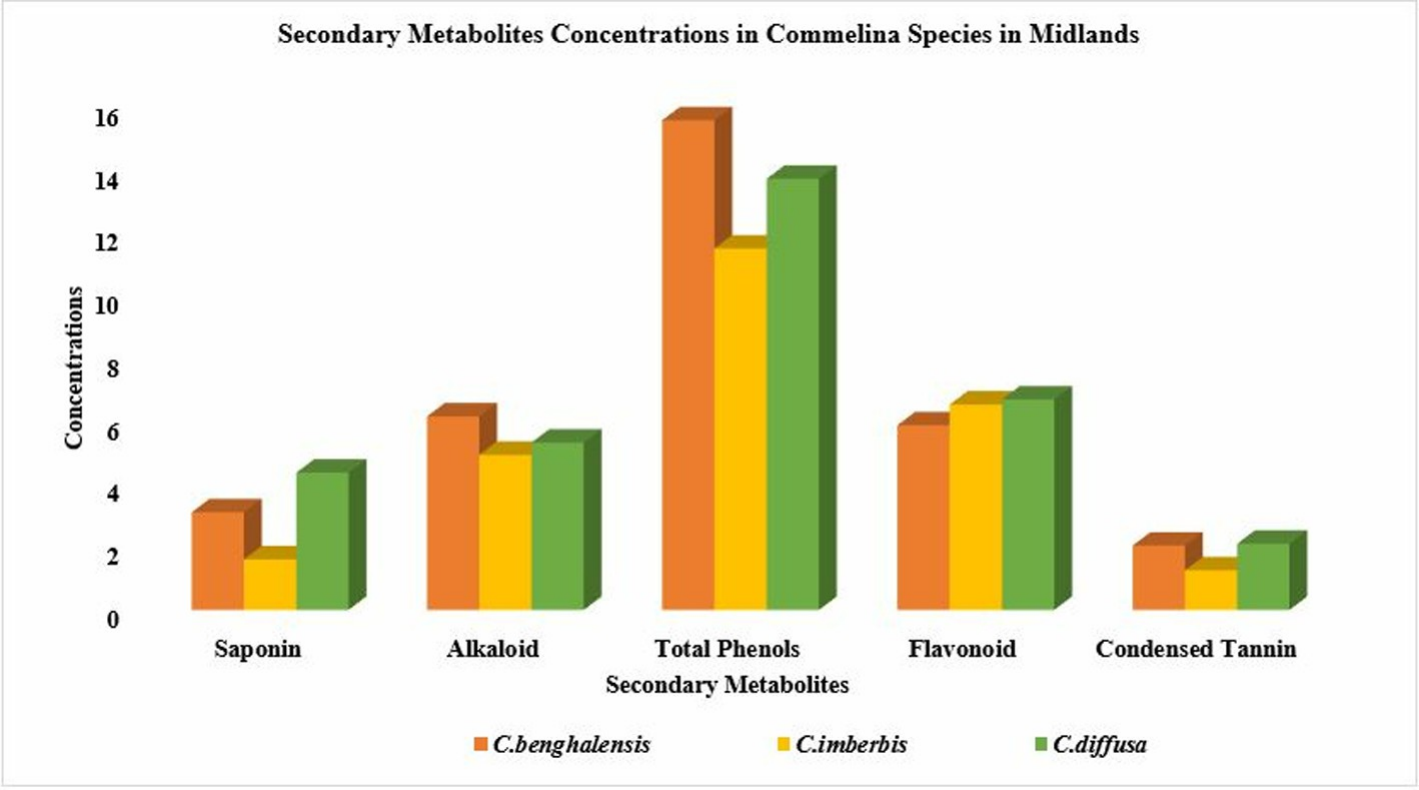
Composition of total phenols in milligram gallic acid equivalents per gram (mg GAE/g), flavonoid and tannin as catechin equivalents (mg CTE/g), saponin (g/Kg) and alkaloids (%) in different *Commelina* species in midlands of Konso zone, southern Ethiopia.

**Fig 4:**
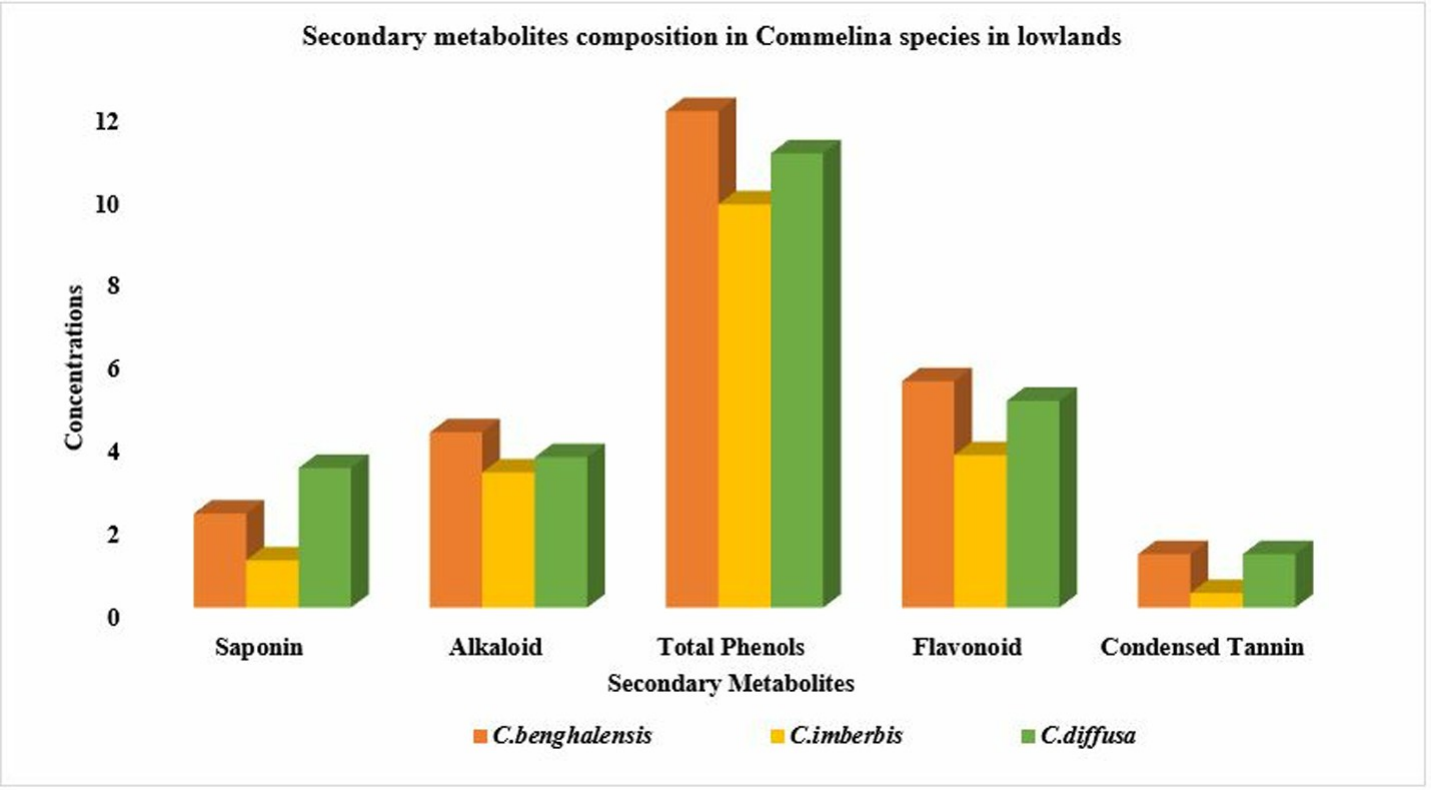
Composition of total phenols in milligram gallic acid equivalents per gram (mg GAE/g), flavonoid and tannin as catechin equivalents (mg CTE/g), saponin (g/Kg) and alkaloids (%) in different *Commelina* species in lowlands of Konso zone, southern Ethiopia.

The condensed tannin contents of *Commelina* species were in the range of 0.35–1.29 mg CE/g in the lowlands, which augmented to 1.26–2.11 mg CE/g in the midlands. In the present study, among species variability in condensed tannin concentration exhibited a consistent pattern with higher values observed equally in *C.benghalensis* and *C.diffusa* and lower values noted in *C.imberbis* at both altitudes. Altitudinal changes in secondary metabolite composition of *Commelina* species in the Konso zone, southern Ethiopia were illustrated in Fig 3 (midland variability) and Fig 4 (lowland variability pattern).

### Correlation between season, altitude and the secondary metabolite examined in *Commelina* species

The correlation patterns among season, altitude and the secondary metabolites are depicted in Table 4. Alkaloid storage in *Commelina* species in the present study had a significant positive correlation (P<0.05) with altitude (r = 0.457), total phenols (r = 0.857), flavonoid (r = 0.90), condensed tannin (r = 0.843) and season (r = -0.791). As presented in Table 4, the total phenols concentration had a highly significant positive correlation (P<0.001) with alkaloids (r = 0.875), flavonoid (r = 0.793), condensed tannin (r = 0.769) and season (r = -0.727). Similarly, condensed tannin concentration showed a significant (P<0.05) positive correlation with altitudinal changes (r = 0.44), seasonal variation (r = 0.702), alkaloids (r = 0.843), total phenols (r = 0.769) and flavonoids (r = 0.802).

**Table 4:** Correlation between season, altitude and the secondary metabolite examined in *Commelina* species.

	Altitude^a^	Season^b^	Saponin (g/Kg)	Alkaloid (%)	Total Phenols (mg GAE/g)	Flavonoid (mg CE/g)
Season	0.00	1				
Saponin (g/Kg)	0.271	-0.579^**^	1			
Alkaloid (%)	0.457^*^	0.791^***^	-0.234	1		
Total Phenols (mg GAE/g)	0.398	0.727^***^	-0.168	0.875^***^	1	
Flavonoid (mg CE/g)	0.401	0.726^***^	-0.207	0.90^***^	0.793^***^	1
Condensed Tannin (mgCE/g)	0.440^*^	0.702^***^	0.015	0.843^***^	0.769^***^	0.802^***^

^a^Altitudinal changes refers to changes from low altitude to mid altitude

^b^Seasonal changes refers to changes from wet season to dry season

* = P<0.05

** = P<0.01

***P<0.001; mg CE = milligram of catechin equivalent; mg GAE = milligram of gallic acid equivalent

## Discussion

### Seasonal variability in the secondary metabolite content of *Commelina* species

Being living things, plants were in a continuous and complex interaction with all entities, be it biotic or abiotic, in their particular environment [28]. It was well established that the secondary metabolites produced by plants themselves were considered as potential mediators of the interactions that exist between plants and their environment. So, changes in the plant secondary metabolites’ concentration were expected as a result of fluctuations in environmental conditions [4]. Furthermore, Kumar and Kumari, [20], pointed out that, with changing seasons, environmental temperatures, UV radiation, salinity, oxygen concentration, and wind velocity were changed leading to fluctuations in secondary metabolite concentration in plants. This corresponds to the significant influence of season on the secondary metabolite concentration of *Commelina* species observed in the present study.

The average concentration of alkaloids observed for *Commelina* species in dry (3.67%) and wet season (7.02%) was definitely higher than the observations of Onyeong et al. [29], who reported alkaloid contents of 2.98% and 1.98% in rainy and dry season for tropical herbs. The authors also noted the saponin contents of the herbs, 1.04 g/Kg in dry season and 0.88 g/Kg in wet season, which were by far lower than 1.28 g/Kg and 2.65 g/Kg saponin concentrations observed in the present study for *Commelina* species in dry season and wet season respectively. The observed disparity in the secondary metabolite composition of different herbaceous species irrespective of seasonal differences could be justified either by the inherent nature of each species or variability in environmental conditions like rainfall distribution patterns, fluctuating temperatures, UV radiation, salinity, oxygen concentration, and wind velocity [2, 20, 21].

Considerable bodies of literature disclose that, besides genetic variability, the growth stage of a given plant also influences the biogenesis of secondary metabolites. The observations of Maknickiene et al., [30] and Fuchs et al., [31], exposed that, the alkaloid content changes constantly in a plant throughout the growth period, and the maximum stocks of alkaloids in leaves are accumulated before flowering. The average contents of total phenol, and total flavonoid and antioxidant activities in secondary metabolite rich plants were also observed to be significantly higher in matured plants than their younger counterparts [32]. Similarly, Adegbaju et al., [33] also argued that, with advancing maturity, the concentration of phenolic compounds increases significantly. Furthermore, the availability of water in the soil was also considered as a known factor related to the variation in the production of metabolites in plants. Plant samples collected during the rainy periods were mostly rich in primary metabolites such as sugars and nucleosides. While samples collected during the dry periods were rich in secondary metabolites like total phenols, alkaloids and flavonoids. In the same way, samples collected during the transition period between the dry and rainy seasons yielded intermediate levels of both primary and secondary metabolites [4, 34]. Consequently, the higher concentrations of total phenols, alkaloids, flavonoids and condensed tannins in *Commelina* species could correspond either to the maturity stage of the plants or to the expected soil water deficiency in the dry seasons of the year [28, 35].

Zhou et al. [34] reported that, the accumulation of total phenols and flavonoids was correlated with adaptability of a given plant species to withstand environmental stresses like draught. So, the higher concentration of total phenols and flavonoids in *C.benghalensis* over *C.imberbis* could imply higher draught resistance potential of *C.benghalensis* was higher than that of *C.imberbis*. Supporting the afore mentioned reflection, Kebede et al. [14] argued that, *C*. *benghalensis* was stronger than *C.diffusa* to maintain its leafy and green vigor long periods after onset of dry periods in a year. Likewise, the comparable accumulation of total phenols and flavonoids in *C.diffusa* and *C.africana* irrespective of season could imply the analogous stress resistance potential of these species.

Alkaloids were known to be a key secondary metabolite responsible for the plants’ therapeutic properties. And, variability in alkaloid accumulation among species elucidates discrepancy in the therapeutic nature of the plants [8, 9]. Consequently, from the observations of the present study, it follows that, all of the *Commelina* species investigated in the present study would have comparable therapeutic potential at dry season. On the contrary, the potential of *Commelina* species to serve therapeutic function in wet season follows the trend that the potential of *C.albescene > C.diffusa > C.imberbis*.

Phrompittayarat et al. [36] argued that, for the saponin rich plant, *Bacopa monnieri*, the higher humidity in the rainy season is suitable for the production of saponins, therefore, high total saponin contents were quantified in the rainy season. Similarly, Akiode et al., [37] reported that, a significantly higher amount of saponin was obtained during the rainy periods of the year from leaves of *Azadirachta indica* which, agrees with the higher saponin content of *Commelina* species in rainy season as compared to dry season. On the contrary to the observations of *Commelina* species and its corresponding findings, Pecetti et al., [38] noted a 46% increase in the saponin content of *Medicago sativa* leaves from wet season to dry season. To mediate the inconsistency among different findings about the effect of seasonal changes on the saponin content of plants, Zhou et al. [34] pointed out that stress conditions like lack of rain or soil humidity significantly induce many biochemical and physiological factors and diverse genetic responses. So, depending on the species or cultivar, diverging quantities of secondary metabolites could be detected in the plants in response to the imposed stress. Moreover, Phrompittayarat et al. [36] claimed that, the saponin content in various plant organs displays dynamic changes in different phases of development and different growth seasons. So, seasonal fluctuation patterns of this metabolite can vary greatly between species, as is exemplified in ageing roots of *Bacopa monnieri*, which display either a significant increase or decrease in the level of saponins.

Condensed tannins (CT) are structural molecules composed of polyphenols, which bind to proteins, metal ions and polysaccharides, such as starch, cellulose, and hemicellulose [39]. In the range of 2–4 mg/g CT can promote beneficial effects, like reducing H_2_ and acetate formation in the rumen, in addition to inhibiting the growth of methanogenic microorganisms, thus reducing the production of enteric CH_4_, improving protein utilization by ruminant animals through preventing excessive rumen degradation of protein and increasing the amount of rumen escape or bypass protein that will be digested in the small intestine [40]. Beyond 6 mg/g of DM in the diet, CT are considered antinutritional factors because they reduce intake, fiber digestibility and animal performance. At CT levels, over 9% of toxic and even lethal effects have been reported in cattle. While sheep and goats had a tolerance threshold up to 9 mg/g [9]. It follows that, all of the *Commelina* species examined in this study contained tannin at levels (0.40–1.73 mg CE/g in wet season and 1.21–3.49 mg CE/g in dry season) tolerable to cattle, sheep and goats, so, animals consuming the species could benefit from the beneficial effect of the tannin contained in them.

Saponin levels in the range of 15–20 g/kg DM were reported to be tolerable for ruminants [41]. Lower levels of saponin have been shown to act as defaunating agents. The defaunation can effectively break protozoan activity and lower acetate production and CH4 emissions [10]. Low levels of saponins increase nutrient digestibility, improve N retention by regulating ammonia concentration [3]. Excess content of saponins in the fodder adversely affects animals, causing a depression in growth rates, inhibiting enzyme activity, and leading to a reduction in nutrient absorption in the digestive tract, as well as that saponins are bitter-tasting molecules [42, 43]. Findings of the present study implies that animals consuming *Commelina* species will be in a potential position to benefit from the positive nutritional influences of saponins in the herb species.

### Altitudinal variability in the secondary metabolite content of *Commelina* species

The significant influence of altitude on the secondary metabolite composition of *Commelina* species observed in this study coincides with the findings of previous researchers [21, 44]. As to Jugran et al. [44], the type and amounts of active constituents in plants vary with geographical locations and altitude due to varied environmental conditions. Changes in altitudinal gradients influence the nature of ecological factors such as soil nutrients, precipitation, and mean temperature, which in turn exert their effect, either direct or indirect, on the secondary metabolite contents and biological activities of the plants. The reasonable increase in the secondary metabolites of *Commelina* species with increasing altitude was also supported by the reflections of Lei et al., [45], who argued that strong ultraviolet radiation and low environment temperature at higher altitudes force plants to produce higher amounts of secondary metabolites as compared to plants growing at lower altitudes to withstand environmental stresses associated with increased elevations. The influence of altitude on accumulation of different secondary metabolites of *Commelina* species observed in this study was also supported by the reports of Priyanka et al. [46], and Aslam et al. [47].

Ultraviolet radiation reaching the Earth’s surface imposes a photo-oxidative stress on plants that influences their physiological activity and morphology [48]. Oxidative stress (OS) is defined as a state of imbalance between oxidation and antioxidation in the body of a living organism, with oxidation tending to prevail, leading to a large production of oxidative intermediate products commonly called free radicals [49]. Unless removed by adaptive mechanisms, these free radicals impose an adverse effect on general plant physiology and function. Iswarya Velu et al., [50] argued that, in response to oxidative stress and the damaging influence of free radicals, plants were observed to produce secondary metabolites like phenolic acids, alkaloids and flavonoids. These compounds were reported to have the capacity of acting as a reducing agent, single oxygen quenchers and hydrogen donors. According to the reflections of Julkunen-Tiitto et al., [51], these phytocompounds filter UV radiation avoiding or minimizing their penetration into internal tissues. Phenolic compounds can also act as antioxidants by scavenging the free radicals produced under oxidative stress [49]. The higher production of secondary metabolites that can scavenge the free radicals may be a part of the defense mechanism of the plant as they counterbalance the effect of the free radicals. They do the free radical counterbalancing action by donating an electron to a charged free radical and terminating the chain reaction before vital molecules are damaged, inhibiting cellular damage [52]. It is well established that UV radiation increases with increasing altitudinal gradients, leading to increased production of secondary metabolites at higher altitudes than at low altitude [21, 44, 45], which qualifies the higher concentration of all of the secondary metabolites examined in *Commelina* species in midlands as compared to lowlands in the present study.

Al-Rowaily et al., [1] investigated the secondary metabolites composition of wild herbs and grasses in Egyptian lowlands and reported the total phenols content of *Lasiurus Scindicus* and *Panicum turgidum* (9.59–10.69 mg GAE/g) comparable with total phenols of *Commelina* species in the lowlands in the present study (9.73–11.98 mg GAE/g). On the other hand, the condensed tannin (0.35–1.29 mg CE/g) and flavonoid (3.68–5.46 mg CE/g) concentration of *Commelina* species observed in lowlands were by far lower than 5.31–18.27 and 4.33–26.10 respectively reported for other lowland herbs [1]. The mean total phenolic concentration of (23.67 mg GAE/g) reported other midland herb species [21] was also higher than the mean midland total phenol concentration of *Commelina* species (13.61 mg GAE/g). This discrepancy in findings could be attributed to very commonly reported cultivar and environmental condition specific differences [2, 45].

Though *C.benghalensis* and *C.diffusa* were observed to have the highest comparable condensed tannin amounts and *C.imberbis* was accumulating the least levels consistently at both altitudes of the study area, none of the examined *Commelina* species accumulated condensed tannin levels that could impose antinutritional effects to reduce voluntary feed intake and nutrient digestibility [9] regardless of altitudinal difference. It was well stablished that, saponin was highly correlated to palatability of a particular plant species to herbivores because they were konown for their bitter taste in nature [42, 43]. Correspondingly, it follows that, irrespective of altitudinal alteration, *C.diffusa* was expected to be highly palatable followed by *C.benghalensis while C.imberbis* would be the least palatable of all.

### Correlation between season, altitude and secondary metabolites examined

The positive significant correlation between the alkaloids and condensed tannin contents of *Commelina* species with increasing altitude was supported by the findings of Rieger et al., [53] and Lianopoulou et al. [54]. The positive correlation of radical scavenging secondary metabolites with increasing altitude is suggestive of the thought that at higher altitudes the plants produce high amounts of secondary metabolites as a defense strategy against low temperature and high UV radiation exposure at higher altitudes [28].

In agreement with the present study, Akiode et al., [37] reported that, there exists a significant negative correlation between saponin content and seasonal changes from wet to dry seasons of the year. On the other side, Nagy and Makleit [55] reported that, no correlation was found between the sampling season and the total saponin content of the samples. The discrepancy in the reports of different scholars with regard to the association between saponin content and seasonal changes could be attributed to the inherent nature of plant species, corresponding to genetic influence, and diverging influence of environmental stress conditions on secondary metabolite composition of plant species [34]. The negative correlation between saponin content of *Commelina* species in the present study could be qualified by the decreasing trend observed in saponin concentration progressing from wet season to dry season (Table 1).

In the present study, the concentrations of total phenols, flavonoids, alkaloids and condensed tannins showed a significant positive relationship with considerable body of literature stands in support of these findings [44, 56, 57] which disclosed the existence of positive significant correlation between total phenol, flavonoid, alkaloids and condensed tannins contents of secondary metabolite rich plant species like *Hedychium spicatum*, *Torilis leptophylla*, *Juglans regia* and *Valeriana jatamansi*. In plant physiology, total phenols, flavonoids, alkaloids and condensed tannins were reported to play concurrent and shared roles, as a defense mechanism, counterbalancing and escaping the influence of biotic and abiotic stresses. And, in response to stressful conditions, the biogenesis and storage of secondary metabolites in plant tissues and organs show a comparable degree of change [28, 34, 49]. So, the shared and cross-matching biological role of the secondary metabolite could rationalize the positive significant correlation observed between the afore mentioned biological molecules examined in *Commelina* species in the present study.

## Conclusions

The secondary metabolite composition of *Commelina* species was reasonably influenced by both seasonal and altitudinal changes. The saponin contents of the herb species were higher in the wet season (2.65 g/Kg) than the dry season (1.28 g/Kg). The mean alkaloid, total phenols, flavonoids, and condensed tannin concentrations in *Commelina* species showed an increasing trend from wet season to dry season. With advancement in altitudinal gradients, from lowland to midland, the values of all of the secondary metabolites examined in the present study, saponin, alkaloids, total phenols, flavonoids and condensed tannins, revealed an increasing pattern. The secondary metabolite concentrations of *Commelina* species were below the levels tolerable for cattle, sheep and goats. Alkaloids, total phenols, flavonoids and condensed tannin concentrations of *Commelina* species showed positive correlations with each other and with seasonal and altitudinal changes also. So, it can be concluded that *Commelina* species could serve as a safe and beneficial forage herb to boost nutrient intake, improve nutrient use efficiency and hinder methane emissions, for animals consuming them, in areas where they were present in abundance.

## Supporting information

S1 Data Word data for all SMS. (DOCX)

S2 Data Standardized figures. (DOCX)
