# Supplementary material for: Seasonal and altitudinal dynamics in secondary metabolite composition of Commelina forage species in Konso zone, southern Ethiopia
Source: PLoS One. 2024 Nov 26;19(11):e0314358. doi: 10.1371/journal.pone.0314358 (PMC11594514; doi:10.1371/journal.pone.0314358)
Supplement: S1 Data — (DOCX) [file pone.0314358.s001.docx]

**Total saponin**

| Sample code | Total Saponin (g/kg of dried sample) | Average |
| --- | --- | --- |
| BngLR | 3.40  3.52 |  |
| DifLR | 4.95  4.78 |  |
| ImbLR | 0.80  0.68 |  |
| ImbLD | 2.00  1.90 |  |
| AfrLR | 1.65  1.50 |  |
| AibLD | 1.25  1.27 |  |
| ImbMR | 2.45  2.51 |  |
| BngMD | 1.85  1.74 |  |
| DifLD | 5.22  5.35 |  |
| AibLR | 0.80  0.72 |  |
| BngLD | 2.70  2.81 |  |
| ImbMD | 0.35  0.30 |  |
| BngMR | 2.72  2.80 |  |
| DifMD | 1.80  1.95 |  |
| AfrLD | 0.20  0.14 |  |
| DifMR | 3.40  3.51 |  |

g/Kg = mg/g

**Total saponin 1**

| Sample code | Total Saponin (g/kg of dried sample) | Average |
| --- | --- | --- |
| BngLR | 3.40  3.52 |  |
| BngLD | 2.70  2.81 |  |
| BngMR | 2.72  2.80 |  |
| BngMD | 1.85  1.74 |  |
| ImbLR | 0.80  0.68 |  |
| ImbLD | 2.00  1.90 |  |
| ImbMR | 2.45  2.51 |  |
| ImbMD | 0.35  0.30 |  |
| DifLR | 4.95  4.78 |  |
| DifLD | 5.22  5.35 |  |
| DifMR | 3.40  3.51 |  |
| DifMD | 1.80  1.95 |  |
| AibLR | 0.80  0.72 |  |
| AibLD | 1.25  1.27 |  |
| AfrLR | 1.65  1.50 |  |
| AfrLD | 0.20  0.14 |  |

**Total saponin Seasonal Variability**

| Sample code | Total Saponin (g/kg of dried sample) | Average |
| --- | --- | --- |
| BngMR | 3.40  3.52 | 3.46 |
| BngMD | 2.70  2.81 | 2.76 |
| BngLR | 2.72  2.80 | 2.76 |
| BngLD | 1.85  1.74 | 1.80 |
| ImbLR | 2.00  1.90 | 1.95 |
| ImbLD | 0.35  0.30 | 0.33 |
| ImbMR | 2.45  2.51 | 2.48 |
| ImbMD | 0.80  0.68 | 0.74 |
| DifLR | 4.95  4.78 | 4.86 |
| DifLD | 1.80  1.95 | 1.87 |
| DifMR | 5.22  5.35 | 5.28 |
| DifMD | 3.40  3.51 | 3.45 |
| AibLR | 1.25  1.27 | 1.25  1.27 |
| AibLD | 0.80  0.72 | 0.80  0.72 |
| AfrLR | 1.65  1.50 | 1.65  1.50 |
| AfrLD | 0.20  0.14 | 0.20  0.14 |

**Total saponin Altitudinal Variability**

| Sample code | Total Saponin (g/kg of dried sample) | Average |
| --- | --- | --- |
| BngMR | 3.40  3.52 | 3.46 |
| BngMD | 2.70  2.81 | 2.76 |
| BngLR | 2.72  2.80 | 2.76 |
| BngLD | 1.85  1.74 | 1.80 |
| ImbLR | 2.00  1.90 | 1.95 |
| ImbLD | 0.35  0.30 | 0.33 |
| ImbMR | 2.45  2.51 | 2.48 |
| ImbMD | 0.80  0.68 | 0.74 |
| DifLR | 4.95  4.78 | 4.86 |
| DifLD | 1.80  1.95 | 1.87 |
| DifMR | 5.22  5.35 | 5.28 |
| DifMD | 3.40  3.51 | 3.45 |

**Total Alkaloid** **Seasonal Variability**

| Code | Total alkaloid (%,w/w) | Average |
| --- | --- | --- |
| BngLR | 3.05  3.20 | 3.12 |
| BngLD | 5.40  5.25 | 5.33 |
| BngMR | 4.60  4.42 | 4.31 |
| BngMD | 7.94  7.66 | 7.80 |
| ImbLR | 1.40  1.53 | 1.46 |
| ImbLD | 5.00  5.11 | 5.56 |
| ImbMR | 2.47  2.65 | 2.56 |
| ImbMD | 7.23  7.40 | 7.32 |
| DifLR | 3.17  3.32 | 3.25 |
| DifLD | 3.90  4.11 | 4.50 |
| DifMR | 3.51  3.39 | 3.45 |
| DifMD | 7.15  7.25 | 7.20 |
| AfrLR | 4.30  4.20 | 4.30  4.20 |
| AfrLD | 8.98  7.44 | 8.98  7.44 |
| AibLR | 5.13  4.95 | 5.13  4.95 |
| AibLD | 7.95  8.10 | 7.95  8.10 |

**Total Alkaloid Altitudinal Variability**

| Code | Total alkaloid (%,w/w) | Average |
| --- | --- | --- |
| BngLR | 3.05  3.20 |  |
| BngLD | 5.40  5.25 |  |
| BngMR | 4.60  4.42 |  |
| BngMD | 7.94  7.66 |  |
| ImbLR | 1.40  1.53 |  |
| ImbLD | 5.00  5.11 |  |
| ImbMR | 2.47  2.65 |  |
| ImbMD | 7.23  7.40 |  |
| DifLR | 3.17  3.32 |  |
| DifLD | 3.90  4.11 |  |
| DifMR | 3.51  3.39 |  |
| DifMD | 7.15  7.25 |  |

**Total phenolics Seasonal Variability**

| Sample code | TPC (mgGAE/g) | Average |
| --- | --- | --- |
| BngLR | 9.997  8.439 | 9.218 |
| BngLD | 14.721  14.755 | 14.738 |
| BngMR | 14.586  14.098 | 14.342 |
| BngMD | 16.902  16.759 | 16.831 |
| ImbLR | 6.182  7.2 | 6.691 |
| ImbLD | 13.66  11.891 | 12.776 |
| ImbMR | 6.619  7.158 | 6.889 |
| ImbMD | 16.38  15.9 | 16.14 |
| DifLR | 7.344  9.651 | 8.498 |
| DifLD | 13.155  13.694 | 13.424 |
| DifMR | 12.767  13.153 | 12.96 |
| DifMD | 14.603  14.376 | 14.489 |
| AlbLR | 8.961  9.752 | 8.961  9.752 |
| AlbLD | 12.767  13.407 | 12.767  13.407 |
| AfrLR | 9.017  11.225 | 9.017  11.225 |
| AfrLD | 12.414  13.694 | 12.414  13.694 |

**Total phenolics Altitudinal Variability**

| Sample code | TPC (mgGAE/g) | Average |
| --- | --- | --- |
| BngLR | 9.997  8.439 |  |
| BngLD | 14.721  14.755 |  |
| BngMR | 14.586  14.098 |  |
| BngMD | 16.902  16.759 |  |
| ImbLR | 6.182  7.2 |  |
| ImbLD | 13.66  11.891 |  |
| ImbMR | 6.619  7.158 |  |
| ImbMD | 16.38  15.9 |  |
| DifLR | 7.344  9.651 |  |
| DifLD | 13.155  13.694 |  |
| DifMR | 12.767  13.153 |  |
| DifMD | 14.603  14.376 |  |

**Total Flavonoid Seasonal Variability**

| **Code** | **TFC (mgCE/g)** | **Average** |
| --- | --- | --- |
| **BngLR** | 4.254  4.362 | 4.308 |
| **BngLD** | 6.487  6.753 | 6.62 |
| **BngMR** | 4.962  5.023 | 4.993 |
| **BngMD** | 6.708  6.832 | 6.77 |
| **ImbLR** | 2.408  2.669 | 2.539 |
| **ImbLD** | 4.6  5.023 | 4.812 |
| **ImbMR** | 3.177  3.131 | 3.154 |
| **ImbMD** | 9.946  9.914 | 9.93 |
| **DifLR** | 4.223  4.985 | 4.604 |
| **DifLD** | 5.1  5.677 | 5.384 |
| **DifMR** | 4.408  4.869 | 4.639 |
| **DifMD** | 8.692  8.831 | 8.762 |
| **AfrLR** | 3.585  4.485 | 3.585  4.485 |
| **AfrLD** | 8.023  8.177 | 8.023  8.177 |
| **AibLR** | 2.846  2.992 | 2.846  2.992 |
| **AibLD** | 9.129  9.171 | 9.129  9.171 |

**Total Flavonoid Altitudinal Variability**

| **Code** | **TFC (mgCE/g)** | **Average** |
| --- | --- | --- |
| **BngLR** | 4.254  4.362 |  |
| **BngLD** | 6.487  6.753 |  |
| **BngMR** | 4.962  5.023 |  |
| **BngMD** | 6.708  6.832 |  |
| **ImbLR** | 2.408  2.669 |  |
| **ImbLD** | 4.6  5.023 |  |
| **ImbMR** | 3.177  3.131 |  |
| **ImbMD** | 9.946  9.914 |  |
| **DifLR** | 4.223  4.985 |  |
| **DifLD** | 5.1  5.677 |  |
| **DifMR** | 4.408  4.869 |  |
| **DifMD** | 8.692  8.831 |  |

**Total Condensed Tannins Seasonal Variability**

| Code | TCT (mgCE/g) | Average |
| --- | --- | --- |
| BngLR | 0.334  0.31 | 0.322 |
| BngLD | 2.196  2.352 | 2.274 |
| BngMR | 1.484  1.464 | 1.474 |
| BngMD | 2.641  2.599 | 2.62 |
| ImbLR | 0.121  0.099 | 0.11 |
| ImbLD | 0.574  0.606 | 0.59 |
| ImbMR | 0.692  0.706 | 0.699 |
| ImbMD | 1.814  1.844 | 1.829 |
| DifLR | 0.841  0.879 | 0.86 |
| DifLD | 1.722  1.704 | 1.713 |
| DifMR | 0.868  0.98 | 0.924 |
| DifMD | 3.289  3.311 | 3.3 |
| AfrLR | 1.724  1.736 | 1.724  1.736 |
| AfrLD | 3.951  3.929 | 3.951  3.929 |
| AibLR | 1.61  1.55 | 1.61  1.55 |
| AibLD | 1.736  1.9 | 1.736  1.9 |

**Total Condensed Tannins Altitudinal Variability**

| Code | TCT (mgCE/g) | Average |
| --- | --- | --- |
| BngLR | 0.334  0.31 |  |
| BngLD | 2.196  2.352 |  |
| BngMR | 1.484  1.464 |  |
| BngMD | 2.641  2.599 |  |
| ImbLR | 0.121  0.099 |  |
| ImbLD | 0.574  0.606 |  |
| ImbMR | 0.692  0.706 |  |
| ImbMD | 1.814  1.844 |  |
| DifLR | 0.841  0.879 | 0.86 |
| DifLD | 1.722  1.704 |  |
| DifMR | 0.868  0.98 | 0.924 |
| DifMD | 3.289  3.311 |  |
