# Supplementary material for: Seasonal and altitudinal dynamics in secondary metabolite composition of Commelina forage species in Konso zone, southern Ethiopia
Source: PLoS One. 2024 Nov 26;19(11):e0314358. doi: 10.1371/journal.pone.0314358 (PMC11594514; doi:10.1371/journal.pone.0314358)
Supplement: S2 Data — (DOCX) [file pone.0314358.s002.docx]

**Figure ** Catechin calibration curve for the determination of total flavonoid content of **********************

**Figure ** Gallic acid calibration curve for the determination of total phenolic content of **********************

**Figure ** Catechin calibration curve for the determination of total tannin content of **********************
